# The use of angiotensin II in distributive shock

**DOI:** 10.1186/s13054-016-1306-5

**Published:** 2016-05-27

**Authors:** Lakhmir S. Chawla, Laurence W. Busse, Ermira Brasha-Mitchell, Ziyad Alotaibi

**Affiliations:** Department of Veterans Affairs Medical Center, Washington DC, USA; Inova Fairfax Hospital, Falls Church, VA USA; Department of Intensive Care Medicine, Prince Sultan Military City, Kingdom of Saudi Arabia

The interest in the use of non-catecholamine vasopressors for the treatment of hypotension and shock has increased in recent years. The use of vasopressin as an adjunctive vasopressor in shock was reinvigorated by Landry and colleagues [1] and then carefully assessed in the Vasopressin versus Norepinephrine Infusion in Patients with Septic Shock (VASST) trial [2]. In a large, international, multi-center trial, vasopressin demonstrated a satisfactory safety profile, but did not show an improvement in survival compared to norepinephrine [2]. In addition to vasopressin, angiotensin II (ATII) has been proposed as a useful vasopressor for the management of shock [3, 4]. The original studies that assessed ATII for the management of shock were conducted decades ago [3, 5]. In those trials, ATII was assessed primarily in head-to-head studies compared to catecholamine vasopressors, and was shown to have comparable vasopressor effect to norepinephrine [5]. Multiple case reports demonstrated the ability of ATII to work effectively as a vasopressor and also showed that ATII could be used in combination with catecholamines. However, ATII has not been subjected to a randomized controlled trial (RCT) and ATII has not been available at the bedside for at least 15 years. ATII has been used extensively in physiology, hypertension, cancer, and pregnancy studies in humans and has a good safety profile.

Recently, we published in *Critical Care* the first RCT of ATII in patients with distributive shock, and showed that a dose of ATII of 5–40 ng/kg/min was associated with improved blood pressure that resulted in significant catecholamine sparing [6]. In that modest-sized study, we noted that 2 of the 10 patients treated with ATII were exquisitely sensitive to ATII. In these two cases, the subjects receiving physiologic doses of ATII were hypertensive despite the discontinuation of their norepinephrine. When ATII was stopped in these two patients, re-initiation of a high dose of norepinephrine (i.e., 0.3 μg/kg/min) was immediately required in order to maintain mean arterial pressure goals. We speculated that the reason for this sensitivity was likely due to premorbid exposure to angiotensin-converting enzyme (ACE) inhibitors prior to the development of shock. Our theory was that if the subjects were previously treated with ACE inhibitors, their ATII Type I receptors would be upregulated, thus making the patient more sensitive to exogenous ATII infusion. However, after a thorough chart review and re-review, we could not document an ACE inhibitor exposure. While it is possible that the ACE inhibitor exposure was present and not documented, there is an alternative explanation which is related to the nature and distribution of ACE. Angiotensin I (ATI) is converted efficiently to ATII almost exclusively in the lung [7]. ACE is an ectoenzyme which is distributed primarily on the pulmonary capillary endothelium [8, 9]. As a consequence, diseases that affect the pulmonary capillary endothelium can disrupt ACE functionality. Acute respiratory distress syndrome (ARDS) is often associated with significant pulmonary endothelial injury [10]. Patients with more severe ARDS have less capacity to convert angiotensin ATI to ATII, and this disturbance is inversely correlated to the severity of ARDS [11]. Upon re-review, we found that the two patients in our study who were exquisitely ATII sensitive had severe ARDS.

Our revised hypothesis is that patients with severe ARDS may have significant pulmonary endothelial injury, which results in either an absolute or relative insufficiency of ATII due to loss of pulmonary ACE. Pre-clinical and human case reports demonstrate that when ATII production is inhibited by ACE inhibition, patients become catecholamine resistant [12]. Thus, patients with ARDS may be at particular risk for ATII insufficiency, which would likely exacerbate existing hypotension. In addition, ATII insufficiency can lead to acute kidney injury due to decreased intra-glomerular pressure. We hypothesize that some patients with shock and ARDS may be at particular risk for a deleterious cascade of events related to ATII insufficiency (Fig. 1).

**Fig. 1:**
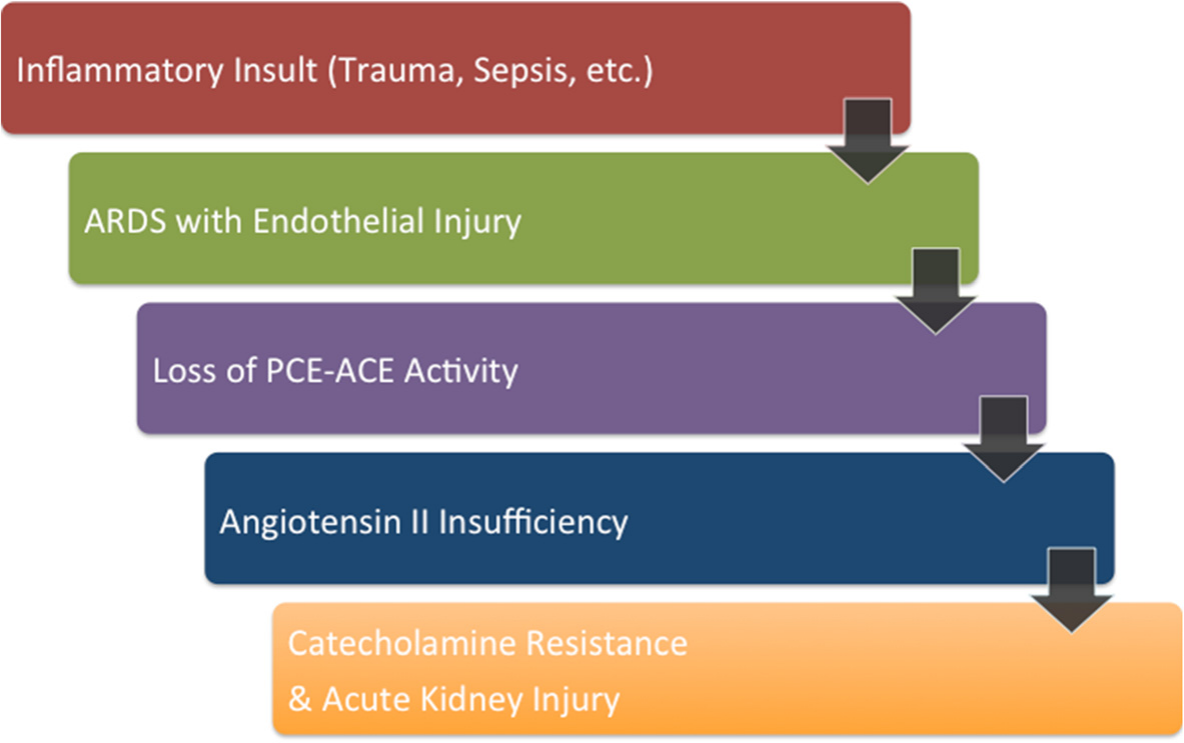
Proposed cascade of events leading to angiotensin II insufficiency. The figure outlines a cascade of events that could occur amongst patients with inflammation and/or lung injury. When acute lung injury is significantly complicated by pulmonary endothelial injury, ACE activity is diminished [11]. Thus, patients who lose ACE activity may be a risk for angiotensin II insufficiency and catecholamine resistance [12]. *ACE* angiotensin-converting enzyme, *ARDS* acute respiratory distress syndrome, *PCE* pulmonary capillary endothelium

We would anticipate that, for those patients with ATII insufficiency, increased levels of ATI and reduced ATII may be indicative of this pathophysiology, and that ATI and ATII levels, as well as the ratio of ATI/ATII, may be useful as biomarkers of early ARDS or ARDS severity prior to the development of severe hypoxemia. Moreover, we would anticipate these patients to be ATII-sensitive. We believe that further research to test this hypothesis is warranted. Currently, ATII is being studied in a multi-center international RCT (NCT02338843) wherein some of these parameters will be assessed and may shed further light on this proposed hypothesis.

## Abbreviations

ACE: angiotensin-converting enzyme
ARDS: acute respiratory distress syndrome
ATI: angiotensin I
ATII: angiotensin II
RCT: randomized controlled trial
VASST: Vasopressin versus Norepinephrine Infusion in Patients with Septic Shock
